# Delivery of paediatric rheumatology care: a survey of current clinical practice in Southeast Asia and Asia-Pacific regions

**DOI:** 10.1186/s12969-021-00498-1

**Published:** 2021-01-23

**Authors:** Sirikarn Tangcheewinsirikul, Swee-Ping Tang, Nicola Smith, Maynart Sukharomana, Sirirat Charuvanij, Soamarat Vilaiyuk, Thaschawee Arkachaisri, Christiaan Scott, Helen E. Foster

**Affiliations:** 1 Division of Rheumatology, Department of Paediatrics, Faculty of Medicine Siriraj Hospital, Mahidol University, Bangkok, Thailand; 2 Paediatric Rheumatology Unit, Selayang Hospital, Selangor, Malaysia; 3 Paediatric Rheumatology, Population Health Sciences Institute, Newcastle University, Newcastle upon Tyne, UK; 4 Division of Rheumatology, Department of Paediatrics, Faculty of Medicine Ramathibodi Hospital, Mahidol University, Bangkok, Thailand; 5 Department of Paediatric Subspecialties, Rheumatology and Immunology Service, KK Women’s and Children’s Hospital, Singapore, Singapore; 6 Division of Paediatric Rheumatology, Department of Paediatrics, University of Cape Town, Red Cross War Memorial Children’s Hospital, Cape Town, South Africa; 7 Newcastle University Medicine Malaysia, Johor, Malaysia

**Keywords:** Paediatric rheumatology, Health care system, SE ASIA/ASIAPAC, Clinical care, Education and training, Medicines, Biological therapies, Workforce

## Abstract

**Background:**

Paediatric rheumatic diseases are a leading cause of acquired disability in Southeast Asia and Asia-Pacific Countries (SE ASIA/ASIAPAC). The aims of this study were to identify and describe the challenges to the delivery of patient care and identify solutions to raise awareness about paediatric rheumatic diseases.

**Methods:**

The anonymised online survey included 27 items about paediatric rheumatology (PR) clinical care and training programmes. The survey was piloted and then distributed via Survey-Monkey™ between March and July 2019. It was sent to existing group lists of physicians and allied health professionals (AHPs), who were involved in the care pathways and management of children with rheumatic diseases in SE ASIA/ASIAPAC.

**Results:**

Of 340 participants from 14 countries, 261 participants had been involved in PR care. The majority of the participants were general paediatricians. The main reported barriers to providing specialised multidisciplinary service were the absence or inadequacy of the provision of specialists and AHPs in addition to financial issues. Access to medicines was variable and financial constraints cited as the major obstacle to accessing biological drugs within clinical settings. The lack of a critical mass of specialist paediatric rheumatologists was the main perceived barrier to PR training.

**Conclusions:**

There are multiple challenges to PR services in SE ASIA/ASIAPAC countries. There is need for more specialist multidisciplinary services and greater access to medicines and biological therapies. The lack of specialist paediatric rheumatologists is the main barrier for greater access to PR training.

**Supplementary Information:**

The online version contains supplementary material available at 10.1186/s12969-021-00498-1.

## Background

Paediatric rheumatic diseases encompass a spectrum of inflammatory conditions including juvenile idiopathic arthritis (JIA). These conditions remain the leading cause of acquired disability in children [1, 2]; risk factors for worse outcome include delay to diagnosis, poor access to appropriate therapies, inadequate specialist services, lack of relevant guidelines being available and children living in countries with worse socioeconomic status [2–4].

There are severe workforce challenges across the globe but especially so in Asia with one paediatric rheumatologist for every 26 million children [2]; this contrast markedly with the recommendations for Europe and North America with one paediatric rheumatologist per 0.42 and 0.25 million children respectively [2]. Although an increase in numbers of rheumatologists and rheumatology trainees were reported [5, 6], there were still limited access to paediatric rheumatologists in Southeast Asia [7]; thus most children with rheumatic diseases were treated mainly by adult rheumatologists and general paediatricians [8, 9].

Currently, several recommendations for standards of care as well as treatment for children with rheumatic diseases have been developed by international paediatric rheumatology associations consisting of experts mainly from high resource income countries (HRIC) [10–18]. These guidelines are not always transferable to clinical practice in middle resource income countries (MRIC) and low resource income countries (LRIC) where there are limited resources and other health care challenges as priorities for health services [19]. The Juvenile Arthritis Management in less resourced countries (JAMLess) recommendations were the first to be aimed at LRIC [19] and highlighted principles to support and develop paediatric rheumatology (PR) including the need to contextually relevant guidance for clinical management, treatments, referrals, monitoring, education and training, advocacy, networks, policy and research. The JAMLess was originally intended for developing recommendations in less resourced countries and focused on JIA, although many of the questions were generic to service delivery in PR [19].

The aims of this study were to build on the previous work from JAMLess [19] to identify and describe the challenges and potential solutions to improve the patient care and raise awareness of paediatric rheumatic diseases in Southeast Asia and Asia-Pacific Countries (SE ASIA/ASIAPAC).

## Methods

The anonymised online survey was developed in collaboration with members of the JAMLess group (CS, HF), using essentially the same questionnaire with 27 items, divided into two major themes; PR awareness-clinical care and PR training programmes among SE ASIA/ASIAPAC. The online survey was piloted and then distributed to clinicians (doctors, nurses, allied health professionals (AHPs)) in the SE ASIA/ASIAPAC regions; recipients were known to be involved in PR clinical care or through general paediatric networks known to have potential to be exposed to children with rheumatic diseases. Participants were sent the link to the survey using existing social media professional groups (WhatsApp™) or by email and were asked to share the link of the survey. No reminders were sent out. The data were collected electronically through the survey online via Survey-Monkey™ between March and July 2019. Anonymity and confidentiality were maintained for all participants throughout the survey.

The survey included questions about the participant (job role, country of work, health care setting, duration of practice, postgraduate PR training, percentage of time devoted to PR patients), opinion about barriers to diagnosis of paediatric rheumatic patients, access to medications including disease modifying antirheumatic drugs (DMARDs) and biological drugs, provision of the multidisciplinary team (MDT) and any additional challenges or barriers affecting clinical care with free text comments. There were also questions about PR training and the type of PR teaching offered (e.g lectures, clinical examination skills and clinical rotation opportunities). Descriptive statistics were used to analyse and present the survey results using collation software provided by Survey-Monkey™.

## Results

**Fig. 1:**
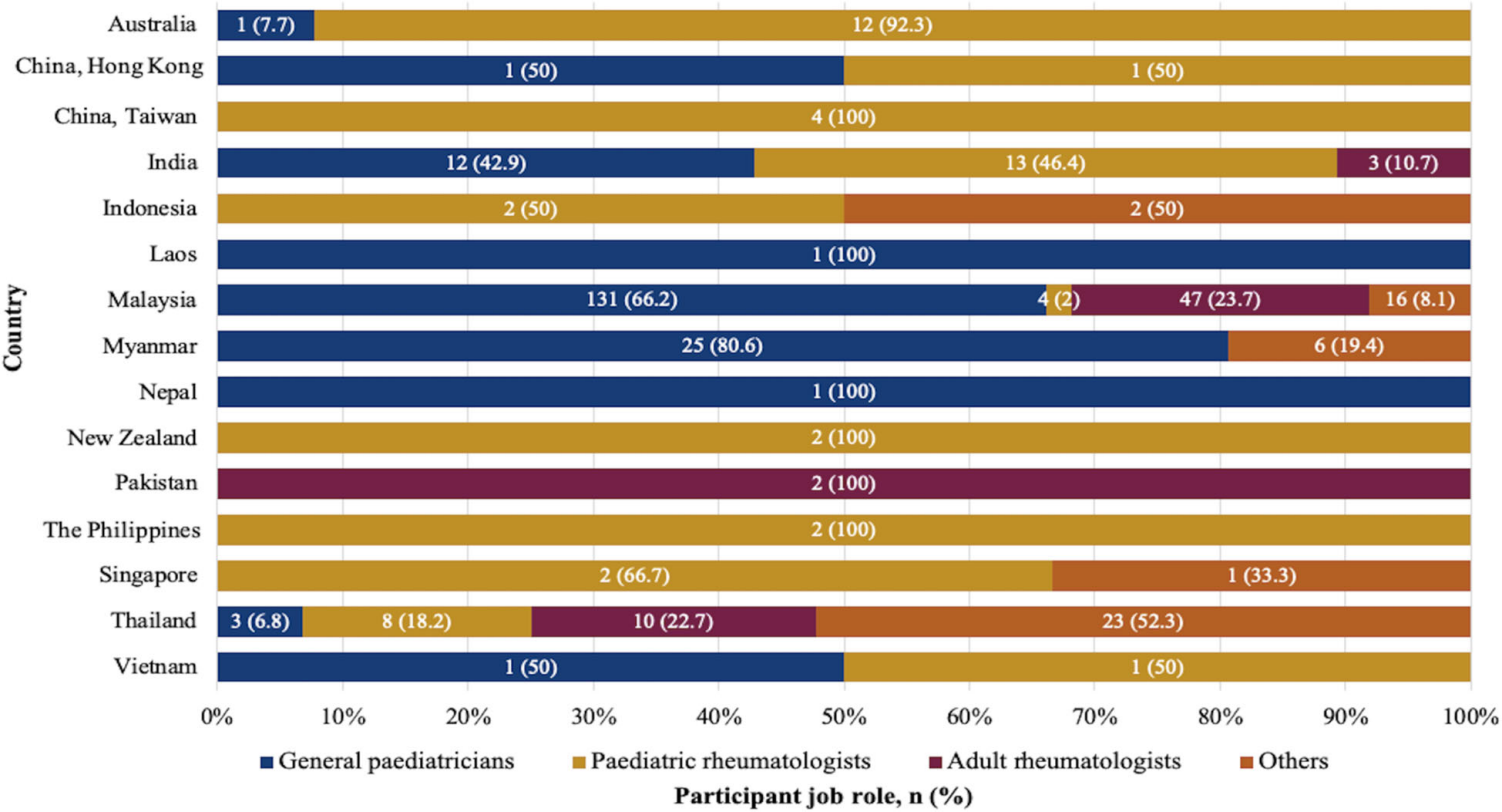
Distribution of the participant job role in each country

There were 340 participants from a total of 14 countries (Fig. 1); the total number of invited participants was unknown so a response rate could not be calculated. The majority of respondents, 261/340 (77.2%) were involved in PR care; most were general paediatricians (52.1%), followed by adult rheumatologists (18.5%), paediatric rheumatologists (15%), and ‘other’ specialists (11.2%; 16 paediatric nephrologists, 5 paediatric allergists, 4 paediatric orthopaedic surgeons, 3 neonatologists, 3 paediatric cardiologists, 1 paediatric haemato-oncologist, 1 paediatric infectious disease specialist, 1 paediatric pulmonologist, and 4 others not identified), 5 general practitioners (1.5%) and others (1.5%); 1 nurse, 2 medical students, 2 medical officers respectively, as shown in Table 1. The term ‘specialist’ is conventionally defined as a physician with specialist certification in their respective country. The majority of participants (59.1%) devoted < 25% of their time to caring children with rheumatic diseases. The duration of clinical practice was broad ranging from < 5 to > 40 years with the majority (27.4%) being 5 to 10 years. Most (41.5%) participants worked in government-funded (public sector) practices, 38.8% in academic centres/ teaching hospitals, and 28.5% in private practice, with the remainder in a mix of state-run/government-funded and private practice. The details of practice settings, number of years in practice and time devoted to PR care as shown in Table 1.

**Table 1:** Demographic data of participants (*N* = 340)

Participants	n (%)
Job roles	
General paediatrician	177 (52.1)
Adult rheumatologist	63 (18.5)
Paediatric rheumatologist	51 (15)
Other specialist^a^	38 (11.2)
General physician	5 (1.5)
Other^b^	5 (1.5)
Unidentified	1 (0.3)
Practice settings	
Public clinical practice	141 (41.5)
Academic centre/ teaching hospital	132 (38.8)
Private clinical practice	97 (28.5)
Mix of government funded and private practice	10 (2.9)
Practice durations, years	
Less than 5	55 (16.2)
5–10	93 (27.4)
10–15	65 (19.2)
15–20	67 (19.8)
20–30	46 (13.6)
30–40	8 (2.4)
More than 40	5 (1.5)
Time devoted to paediatric rheumatology patient care	
Less than 25%	195 (59.1)
26–50%	35 (10.6)
51–75%	7 (2.1)
More than 75%	33 (10)
Unidentified	60 (18.2)

^a^Included 16 paediatric nephrologists, 5 paediatric allergists, 4 paediatric orthopaedic surgeons, 3 neonatologists, 3 paediatric cardiologists, 1 paediatric haemato-oncologist, 1 paediatric infectious disease specialist, 1 paediatric pulmonologist, and 4 others unidentified

^b^Included 1 nurse, 2 medical students, and 2 medical officers

### Paediatric rheumatology awareness and clinical care delivery

**Fig. 2:**
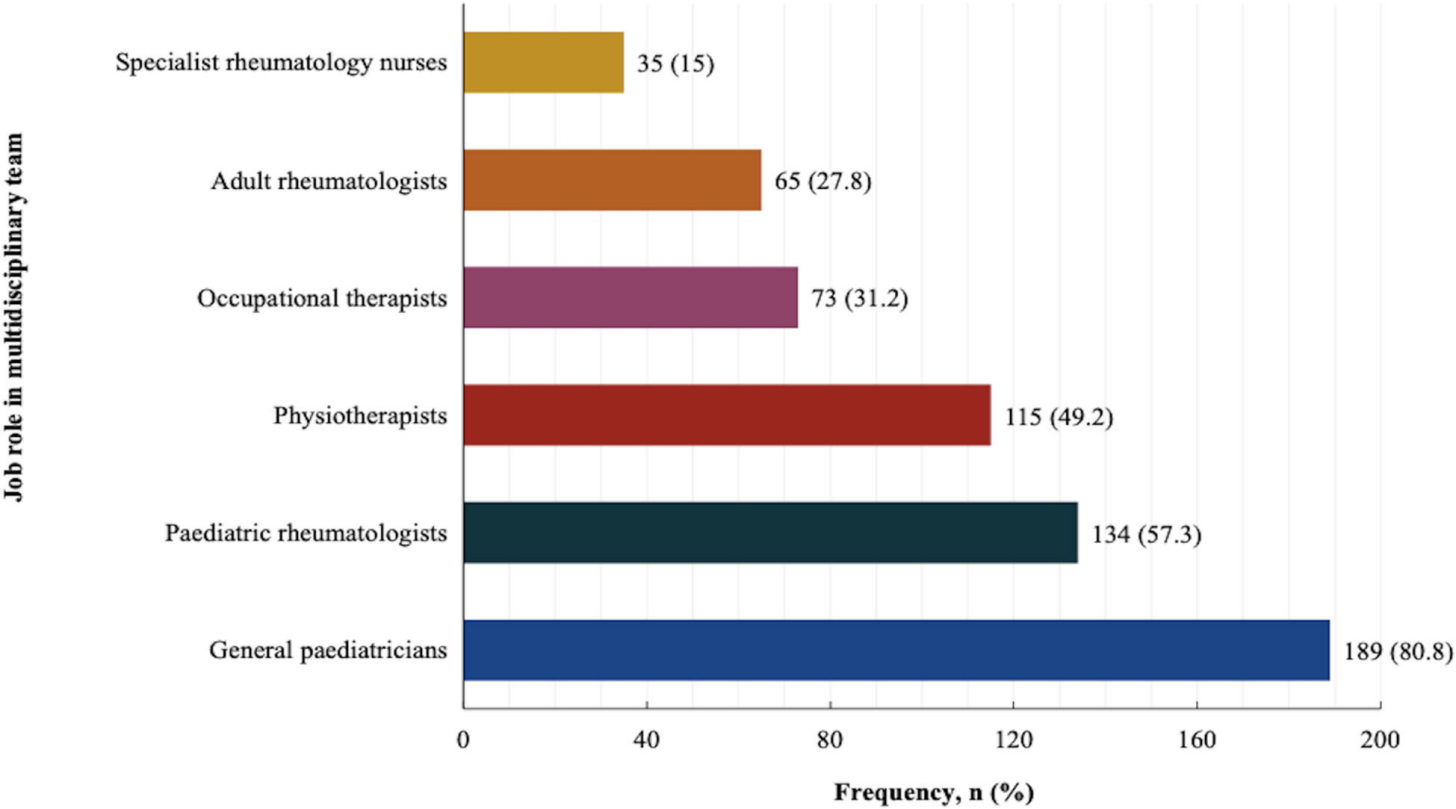
Multidisciplinary team involved in care of children with rheumatic disease

Participants reported that paediatric rheumatologists mainly cared for children with rheumatic diseases (44.9%), followed by general paediatricians (38%) and adult rheumatologists (8.1%). The MDT members involved in patient care included general paediatricians (80.8%), paediatric rheumatologists (57.3%), physiotherapists (49.2%), occupational therapists (31.2%), adult rheumatologists (27.8%) and specialist rheumatology nurses (15%) as shown in Fig. 2.

**Fig. 3:**
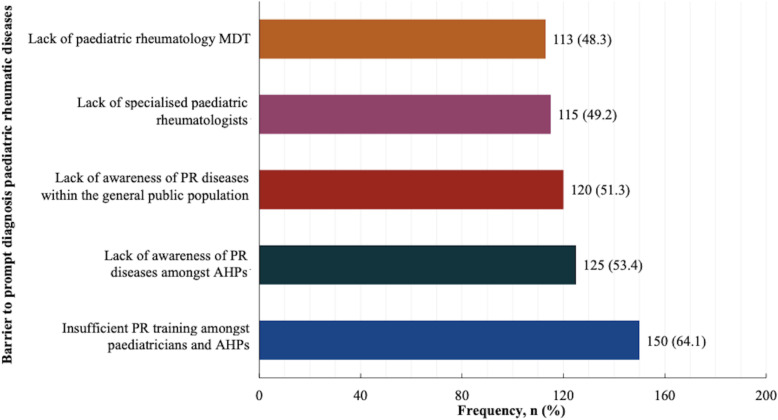
Perceived main barriers to prompt diagnosis paediatric rheumatic diseases. AHPs: Allied health professionals, MDT: Multidisciplinary team, PR: Paediatric rheumatology

The perceived five main barriers to prompt diagnosis of paediatric rheumatic diseases were: 1) insufficient training about childhood rheumatic diseases amongst paediatricians and other AHPs (64.1%): 2) lack of awareness of paediatric rheumatic diseases amongst AHPs (53.4%): 3) lack of awareness of paediatric rheumatic diseases within the general public population (51.3%): 4) lack of specialised paediatric rheumatologists to refer patients to (49.2%): and 5) lack of paediatric rheumatology MDT (48.3%) as shown in Fig. 3.

The main barriers to providing a specialised multidisciplinary PR service in the clinical settings were the absence or inadequacy of provision of specialists (68.2%), the absence or inadequacy of provision of AHPs (49.8%) and financial constraints (43.8%).

There was very variable access to medications in the countries represented in the survey as shown in Tables 2 and 3. Most countries (with the exception of Laos) had access to DMARDS, parenteral corticosteroids and intra-articular steroids (albeit different corticosteroid preparations available). Access to biological therapies was very variable with Singapore having access to all biologics and many other countries having access to few or none (e.g. Indonesia, Laos, Vietnam and Nepal). There was generally very low accessibility to biosimilars; availability of tumour necrosis factor biosimilars (Etanercept, Infliximab) and rituximab biosimilar were reported in Australia, India, Malaysia, Pakistan, the Philippines, Singapore and Thailand whilst other countries reported having access to none. The majority of participants reported financial constraints (62.1%) being the main barrier to accessing biological drugs for patients even if they were available in their countries, followed by non-availability of biological drugs (37.1%) and absence of appropriate specialists to prescribe biological drugs (34.4%).

**Table 2:** Availability of non-biological drugs^a^

Country (n)^b^	Australia (13)	China, Hong Kong (2)	China, Taiwan (4)	India (28)	Indonesia (4)	Laos (1)	Malaysia (198)	Myanmar (31)	Nepal (1)	New Zealand (2)	Pakistan (2)	The Philippines (2)	Singapore (3)	Thailand (44)	Vietnam (2)
Medication															
NSAIDs	√	√	√	√	√	√	√	√	√	√	√	√	√	√	√
Azathioprine	√	√	√	√	√	–	√	√	√	√	√	√	√	√	√
Cyclophosphamide	√	√	√	√	√	–	√	√	√	√	√	√	√	√	√
Cyclosporine/ ciclosporin	√	√	√	√	√	√	√	√	√	√	√	√	√	√	√
Gold	√	√	–	√	–	–	√	√	–	–	–	–	–	√	–
Hydroxychloroquine	√	√	√	√	√	√	√	√	√	√	√	√	√	√	√
Leflunomide	√	√	√	√	–	–	√	√	–	√	√	–	√	√	√
Methotrexate	√	√	√	√	√	√	√	√	√	√	√	√	√	√	√
Sulfasalazine	√	√	√	√	√	–	√	√	√	√	√	√	√	√	√
Oral corticosteroids	√	√	√	√	√	√	√	√	√	√	√	√	√	√	√
Parenteral corticosteroids	√	√	√	√	√	–	√	√	√	√	√	√	√	√	√
Triamcinolone Hexacetonide (Intra-articular corticosteroid)	√	√	√	√	√	–	√	√	√	√	–	√	√	–	–
Other Intra-articular corticosteroids (e.g. Triamcinolone Acetonide, Methylprednisolone Acetate)	√	√	√	√	√	–	√	√	√	√	√	√	√	√	√

^a^The data obtained during the study period

^b^The number of responses per country, 3 missed

**Table 3:** Availability of biological drugs^a^

Country (n)^b^	Australia (13)	China, Hong Kong (2)	China, Taiwan (4)	India (28)	Indonesia (4)	Laos (1)	Malaysia (198)	Myanmar (31)	Nepal (1)	New Zealand (2)	Pakistan (2)	The Philippines (2)	Singapore (3)	Thailand (44)	Vietnam (2)
Medication															
Adalimumab	√	√	√	√	–	–	√	–	–	√	–	√	√	–	√
Etanercept	√	√	√	√	–	–	√	√	–	√	√	√	√	√	–
Golimumab	–	√	–	–	–	–	√	–	–	–	–	√	√	√	–
Infliximab	√	√	√	√	–	–	√	√	–	√	√	√	√	√	–
Anakinra	√	–	√	√	–	–	√	√	–	√	–	–	√	–	–
Canakinumab	√	√	√	–	–	–	√	–	–	–	–	–	√	–	–
Tocilizumab	√	√	√	√	–	–	√	√	–	√	√	√	√	√	√
Belimumab	–	–	–	–	–	–	√	–	–	–	–	–	√	–	–
Rituximab	√	–	–	√	–	–	√	–	–	√	√	√	√	√	–
Abatacept	√	√	–	–	–	–	–	–	–	–	–	–	√	–	–
Secukinumab	–	–	–	√	–	–	–	–	–	–	–	√	√	√	–
Tofacitinib	√	–	–	–	–	–	√	–	–	–	–	√	√	√	–
Ustekinumab	–	–	–	–	–	–	–	–	–	–	–	√	√	√	–

^a^The data obtained during the study period

^b^The number of responses per country, 3 missed

**Fig. 4:**
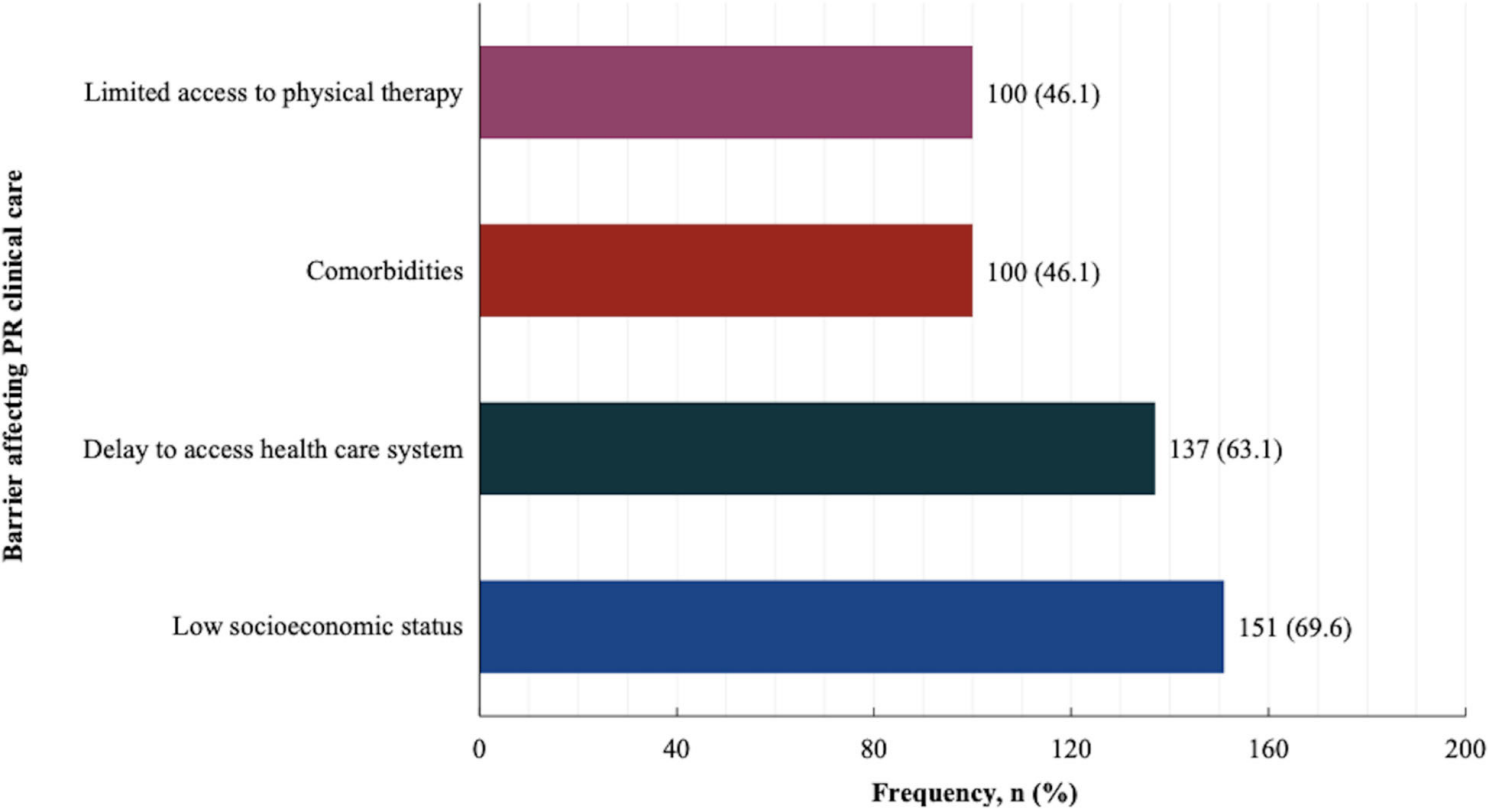
Perceived main challenges affecting paediatric rheumatology clinical care. PR: Paediatric rheumatology

The main challenges affecting clinical care included 1) low socioeconomic status (69.6%), 2) a general delay to access health care system (63.1%), 3) comorbidities such as infection burden (46.1%) and limited access to physical therapy such as physiotherapy and occupational therapy (46.1%), as shown in Fig. 4.

Finally, all participants were asked to share their opinions on how to improve PR clinical care delivery in their setting. Most agreed that there was need for more paediatric rheumatologists (84.9%), specialist therapists (74.8%) and rheumatology nurses (65.1%) as well as more paediatric MSK training programmes for paediatricians and family medicine physicians (73.4%) to raise awareness and facilitate diagnosis and referral.

### Paediatric rheumatology training

**Fig. 5:**
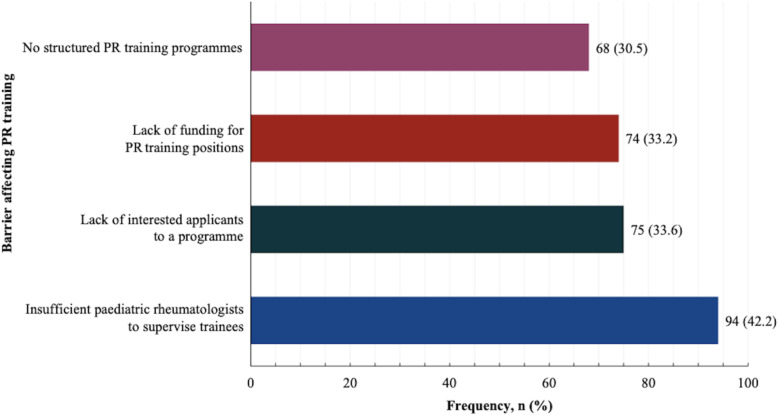
Perceived main challenges affecting paediatric rheumatology training. PR: Paediatric rheumatology

From this survey there appear to be limited opportunities for PR education including lectures, teaching of MSK examination skills and clinical rotation opportunities at the teaching hospitals or universities at both undergraduate and postgraduate levels. There was very low training especially to nurses, AHPs as well as adult rheumatology and general practice trainees. In terms of postgraduate training, the main perceived barriers to PR training were lack of a critical mass of trained paediatric rheumatologists to supervise trainees (42.2%), lack of interested applicants to the programme (33.6%) and lack of funding for PR training positions (33.2%) as shown in Fig. 5. These barriers led to no existing PR training programme for 62.6% of the participants in the country where they were in clinical practice.

## Discussion

This is the first survey describing PR clinical care and training in SE ASIA/ASIAPAC regions and highlighted multiple challenges. Our results demonstrated the paucity of trained paediatric rheumatologists and specialist MDTs as the main perceived barrier to improving PR clinical care. Paediatric rheumatologists also have key roles in education and training leadership, advocacy, policy development and research which are likely to impact on clinical care capacity building. Previous studies from mostly HRIC and MRIC regions demonstrate the global scarcity of paediatric rheumatologists [2, 5, 6, 20–22]; the scarcity is most severe in Africa and Asia, unfortunately in the most populous countries and where there are large numbers of children affected [23].

More PR training programmes are needed [7] and there is need to encourage and support paediatricians to further their training in PR although remains a major challenge given other health care priorities and the lack of paediatricians in many LRIC/MRIC countries [24]. It is therefore imperative for greater efforts to increase awareness and knowledge about PR amongst general paediatricians, other doctors (orthopaedic surgeons, adult rheumatologists), nurses and AHPs who may be the first health care professionals to encounter children with potential rheumatic diseases [19]. Such health care professionals need targeted education and training relevant to their clinical context to enable them to make an accurate diagnosis, be involved in patient care and refer to specialists where available. The PR training is currently developing in some SEA countries, namely Singapore, Malaysia, the Philippines and Thailand. Furthermore, there is need to increase awareness in the general population to encourage early presentation to health care through campaigns (e.g World Young Rheumatic Disease (WORD) Day; https://wordday.org) [25].

The other main perceived barrier to clinical care in SE ASIA/ASIAPAC was affordability, availability and access of medicines (DMARDs and biologics). The variation in availability of biologics in our survey was notable and even in countries regarded as HRIC (e.g Australia and New Zealand). Financial constraints, absence of specialists to supervise the use of biological drugs in clinical practice and drug unavailability are all major barriers to the use of these medicines. The prescribing and monitoring of biological therapies for children with rheumatic diseases is recommended to be under the supervision of specialists [15–17, 19]; therefore, a paucity of specialists is likely a major barrier to access such therapies. Limited access and availability of conventional DMARDs, intraarticular corticosteroids and biological drugs have been reported in other LRIC [9, 26]. The important role of the WHO Essential Medicines List (EML) and need to include medicines used in PR care has been highlighted [27]; revision of the EML is a priority for the PR community to address and if successful, will hopefully improve access to these medicines in many LRIC.

Recommendations and guidelines for clinical care are important levers for change but have been mainly developed within HRIC [10–18] and not transferable to LRIC in the context of other health challenges, poverty and burden of infection [19]. The JAMLess recommendations [19] are the first of their kind for LRIC and most respondents to their surveys were from Africa, Asia and South America. Broadly speaking, our results from SE ASIA/ASIAPAC are similar to those reported in the JAMLess survey; highlighting need for workforce capacity building, greater access to PR training, specialist care and medicines, and targeted educational programmes to raise awareness [28–31]. The SE ASIA/ASIAPAC region has wide diversity in terms of socioeconomic status, population density, disease burden and health care systems. The JAMLess recommendations are likely broadly applicable to SE ASIA/ASIAPAC but more work is needed to produce contextually relevant clinical guidelines.

There are limitations in our study. First, there is likely a selection bias of participants in the survey. Due to the paucity of paediatric rheumatologists in many of the countries surveyed, there were unequal distribution in the responses across some countries. Our survey study was not a population-based study. We sent the link of questionnaires through paediatric networks with potential to reach clinicians involved in clinical care for children with rheumatic diseases. Most responders were from Malaysia, Myanmar and Thailand. It was challenging to involve all hospitals in each country but we believe that as a minimum, tertiary care centres in all the participating countries were represented. Additionally, our survey data found that 223 out of 340 participants responded the question relating to training in PR. The other (117) responders skipped this question. We assumed that the 223 responders had awareness of training in PR in their respective countries and of these, 51 (22.9%) were paediatric rheumatologists. Second, the online survey was in English version and was sent through email and WhatsApp™ so we were unable to ascertain the response rate. Thirdly, areas with dedicated PR care are probably more likely to have responded to this survey and countries without dedicated PR care are inevitably less well represented.

## Conclusions

To the best of our knowledge, this is the first survey of PR clinical care and training in SE ASIA/ASIAPAC and highlights multiple challenges. The Paediatric Global Musculoskeletal Task Force [32] has recently published a ‘Call to Action’ [33] and increasing public and government awareness is important. Facilitating and leveraging change needs support and action from health authorities, higher education institutions and policy makers. We hope that this survey is the initial step for further collaborative working to address many of these challenges and ultimately improve the quality of care for children with rheumatic diseases in the region.

## Supplementary Information

**Additional file 1.**

## Data Availability

Not applicable

## Abbreviations

AHPs: Allied health professionals
ACR: American College of Rheumatology
DMARDs: Disease modifying antirheumatic drugs
EML: Essential Medicines List
EULAR: European League Against Rheumatism
HRIC: High resource income countries
JAMLess: Juvenile Arthritis Management in less resourced countries
LRIC: Low resource income countries
MDT: Multidisciplinary team
MSK: Musculoskeletal
MRIC: Middle resource income countries
NSAIDs: Nonsteroidal anti-inflammatory drugs
PR: Paediatric rheumatology
SHARE: Single Hub and Access point for paediatric Rheumatology in Europe
SE ASIA/ASIAPAC: Southeast Asia and Asia-Pacific Countries
