# Supplementary material for: Delivery of paediatric rheumatology care: a survey of current clinical practice in Southeast Asia and Asia-Pacific regions
Source: Pediatr Rheumatol Online J. 2021 Jan 23;19:11. doi: 10.1186/s12969-021-00498-1 (PMC7824936; doi:10.1186/s12969-021-00498-1)
Supplement: Supplementary file 1 — Additional file 1. [file 12969_2021_498_MOESM1_ESM.docx]

This survey has been developed on behalf of the Paediatric Task Force to improve the lives of children with musculoskeletal conditions around the world.

The Paediatric Task Force is working in collaboration with the Global Alliance for Musculoskeletal Health (<http://bjdonline.org/musculoskeletal-problems-in-children-and-young-people/>), the Paediatric Rheumatology European Society (<https://www.pres.eu>) and Paediatric Musculoskeletal Matters (<http://www.pmmonline.org>). We would be very grateful if you would complete this short questionnaire. Your responses will help give us more information about delivery of paediatric rheumatology care in your setting and country. By the term ‘paediatric rheumatology’ we refer to the care of children and young people with inflammatory rheumatic diseases. There are no right or wrong answers! The information that you provide will help to describe the current delivery of paediatric rheumatology clinical care and give insight as to some of the challenges that need to be addressed. We aim to collate the responses into a report to be published in due course and which we will share with you if you provide us with your email at the end of the questionnaire.

**A. TELL US A LITTLE ABOUT YOU AS A RESPONDER TO THE SURVEY**

1. I look after children with suspected or known rheumatic diseases in my setting

O Yes (If yes please continue to complete the following questions)

O No (If no please skip to the end)

2. Job Role

O Paediatric rheumatologist

O Adult rheumatologist

O General paediatrician

O General practitioner

O Allied health professional – and please specify __________ (e.g. nurse, physiotherapist, occupational therapist)

O Other specialists (please specify, e.g., paediatric/ adult nephrologist, allergist, immunologist, orthopaedic surgeon, cardiologist) __________

3. I practice in (please state your city and country of practice): __________

4. I practice in (select all that apply):

O An academic centre/ teaching hospital

O Private clinical practice

O Government funded (public sector) clinical practice

O A mix of state-run/government funded and private practice

5. Numbers of years I have been in practice (year):

O <5 O 20-30

O 5-10 O 30-40

O 10-15 O 40+

O 15-20

6. In relation to my postgraduate paediatric rheumatology training:

O This was in my home country

O This was in another country (please specify): __________

O This was in a combination of my home country and another country

O I have not had any postgraduate training in paediatric rheumatology

7. Percentage of time I devote to care of paediatric rheumatology patients:

O <25% O 26-50%

O 51-75% O >75%

**B. AWARENESS AND CLINICAL CARE DELIVERY**

8. The main barriers to prompt and correct diagnosis of patients with rheumatic disease in my setting are (select all that apply):

O Complex referral pathways which include professionals who may have limited knowledge of rheumatic diseases

O Lack of awareness of paediatric rheumatic diseases amongst health professionals

O Lack of awareness of paediatric rheumatic diseases within the general public population

O Social and cultural beliefs amongst the general population influencing their presentation to health care

O Insufficient training about rheumatic diseases amongst paediatricians and other health professionals who may see children with suspected rheumatic disease

O Lack of specialist paediatric rheumatologist

O Lack of paediatric rheumatology teams (i.e. doctor, nurse, physiotherapist)

O Lack of access to available international or national guidelines to guide practice

O Lack of a patient/ family advocacy group/organisations to help raise awareness

O Lack of government support to fund clinical services and treatments

O Not applicable

O Other barriers (please specify): __________

9. In my country, children with rheumatic disease are in the main, cared for by: (please tick most relevant)

O General paediatricians

O General practitioners

O Adult rheumatologists

O Paediatric rheumatologists

O Allergists / immunologists

O Not applicable

O Other (specify): __________

10. The usual team members who care for paediatric rheumatology patients in my setting include the following (select all that apply):

O General paediatricians

O Specialised rheumatology nurse(s)

O Paediatric rheumatologist(s)

O Physical therapist(s)

O Occupational therapist(s)

O Adult rheumatologist(s)

O Not applicable

O Other (please specify): __________

11. Drugs available for the treatment of childhood rheumatic disease in my country are (select all that apply):

O NSAIDs O Cyclosporine/ ciclosporin

O Methotrexate O Cyclophosphamide

O Sulfasalazine O Leflunomide

O Gold O Penicillamine

O Hydroxychloroquine O Azathioprine

O Oral corticosteroids

O Parenteral corticosteroids

O Triamcinolone Hexacetonide (Intra-articular corticosteroid)

O Triamcinolone Acetonide (Intra-articular corticosteroid)

12. Biologicals (please specify which ones of the following list):

O Adalimumab O Etanercept

O Infliximab O Rituximab

O Anakinra

O Other biologicals (please specify) __________

13. Biosimilars (if yes, please specify which ones) __________

14. Other drugs available (please specify): __________

15. Other drugs that you ideally would use but are not available (Please specify): __________

16. Barriers to providing a specialised multidisciplinary paediatric rheumatology service in my setting include (select all that apply):

O Absence / inadequate provision of specialists

O Absence / inadequate provision of allied health professionals

O Need to focus on high workload in other areas of my clinical practice with inadequate time for paediatric rheumatology

O Absence / inadequate referral pathways into the service from other specialists or centres

O Absence of recognised centres for patient care

O Geographical

O Financial (please specify): __________

O Cultural (please specify): __________

O There are no barriers to accessing specialised care in my setting

17. Please specify/ explain: __________

18. Barriers to accessing biological therapy in my setting include (select all that apply):

O Absence of specialists who are able to prescribe biologicals

O Absence of biologicals being available

O Absence of approved guidelines

O Absence of policies advocating the use of such therapies in childhood rheumatic diseases

O Financial restraints (please specify): __________

O Cultural factors (please explain): __________

O Administrative (governmental requirements) - please explain): __________

O There are no barriers to biologics in my setting

19. Please specify/ explain: __________

**C. PAEDIATRIC RHEUMATOLOGY TRAINING**

20. The following are barriers to paediatric rheumatology training in my country (select all that apply):

O No structured paediatric rheumatology training programme

O Lack of funding for paediatric rheumatology training positions

O Lack of interested applicants to a programme

O Increased interest for training/ higher priority in resources allotment in other specialties reflecting higher burden of other diseases e.g. infectious disease

O Lack of a critical mass of trained paediatric rheumatologists to supervise trainees

O Patient volume (not enough patients) to justify training programmes

O No positions/ jobs once training is completed

O No recognition of the subspecialty by professional associations or government

O Other (please specify): __________

21. At the teaching hospital/universities in my setting there is paediatric rheumatology teaching included in some form (e.g. lectures, musculoskeletal examination skills, clinical rotation opportunities) for the following (select all that apply)

O For medical students

O Paediatrics residents

O Family medicine residents

O Adult rheumatology residents

O Paediatric rheumatology residents

O Rehabilitation medicine residents

O Nursing students

O Physiotherapy students

O Occupational therapy students

O I don’t know

O Please give further details to describe teaching available __________

**D. MOVING FORWARDS**

22. In your opinion, what are the main challenges /factor(s) affecting the clinical care for children and young people with rheumatic diseases in your setting? (select all that may apply)

O Socioeconomic/ poverty

O Comorbidities (e.g. infection burden)

O Poor/delayed access to the health system

O Cultural issues impacting adherence to appropriate therapy (e.g. medicines, physical therapy)

O Poor access to medicines

O Poor access to physical therapy

O Poor access to hospital-based services

O Poor transitional care to adult rheumatology

O Other (specify): __________

23. What do you think would make a difference (in a helpful way) to the way that you can deliver clinical care in your setting? (select all that may apply)

O More specialists (in paediatric rheumatology)

O More specialist nurses (in paediatric rheumatology)

O More specialist therapists (in paediatric rheumatology)

O More training programmes (in paediatric rheumatology)

O More paediatric musculoskeletal training programmes for paediatricians and family medicine doctors

O Continuing Medical Education programmes to improve and maintain knowledge

O Availability of online or e-learning activities

O Availability of specialist opinion through information technologies (e.g. tele-health)

O Improved access to and affordability of medicines (e.g. biologicals)

O Greater use of information technology (IT) (e.g. apps, tele-health and e-learning) to support clinical care and training

O National guidelines and recommendations relevant to my setting

O Clearer pathways of care relevant to my setting

O More research to inform clinical care in my setting

O More clinical trials of medicines used in my clinical setting

O Patient advocacy groups

O Improved awareness amongst policy makers and politicians

O Other (please describe): __________

24. Please add any other comments or feedback:

25. Do you have a database of patients in your clinical practice?

O Yes

O No

26. Would you like to discuss collaborative research in the future? (be open to us contacting you in the future to discuss collaborative research)?

O Yes

O No

O Not applicable

27. Thank you for your time to complete this questionnaire. If you would like to receive a copy of the summary of the final report, please let us know and provide your contact details: __________
